# Defining the null hypothesis

**DOI:** 10.1186/s12915-015-0181-x

**Published:** 2015-08-29

**Authors:** Emma Saxon

**Affiliations:** BMC Biology, BioMed Central, 236 Gray’s Inn Road, London, WC1X 8HB UK

## Abstract

Virus B is a newly emerged viral strain for which there is no current treatment. Drug A was identified as a potential treatment for infection with virus B. In this pre-clinical phase of drug testing, the effects of drug A on survival after infection with virus B was tested. There was no difference in survival between control (dark blue) and drug A-treated, virus B-infected mice (green), but a significant difference in survival between control and virus B-infected mice without drug treatment (light blue, z-test for proportions *P* < 0.05, n = 30 in each group). The authors therefore concluded that drug A is effective in reducing mouse mortality due to virus B.

## Comment

**Fig. 1.**
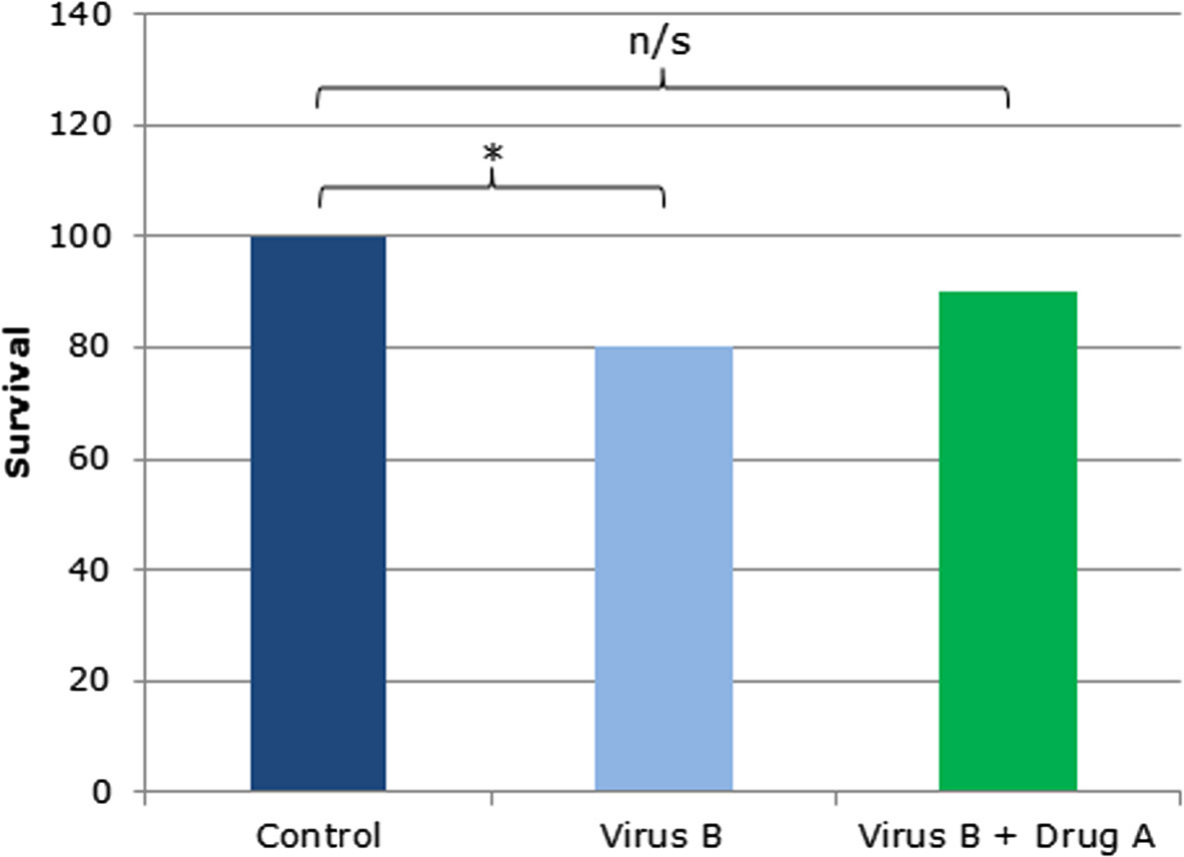
The effects of drug A on the relative survival of mice infected with virus B. Relative survival is significantly decreased in infected mice (light blue), but not in infected mice treated with drug A (green), compared with the control (dark blue); n = 30, z-test for proportions *P < 0.05. *n/s* not significant

Some studies report conclusions based on a null hypothesis different from the one that is actually tested. In this example, the authors tested the effect of a novel antiviral drug on mouse survival 7 days after infection with a virus. The virus alone reduced mouse survival (the light blue bar in Fig. 1, z-test *P* < 0.05), but there was no significant difference between uninfected, untreated control mice (dark blue) and infected, drug A-treated mice (green), so the authors concluded that the drug significantly increased the survival time of infected mice.

But the statistical test used to support the claim was applied inappropriately. In order to conclude that the drug increased the survival of infected mice, the authors would have had to compare infected treated mice (green) with infected untreated mice (light blue), and not with uninfected mice (dark blue). Their results do show that the survival of virus-infected mice was significantly lower than that of uninfected control mice, by 20 %. But the difference between infected untreated and infected treated mice (the light blue versus green bars in Fig. 1, the correct comparison for testing the drug effect) is only 10 %: as the non-significant difference in survival between uninfected control (dark blue) and infected drug-treated mice (green) was also 10 %, it, too, will be non-significant. In this case, the data support the null hypothesis, contrary to the authors’ conclusions.

Note also that the effects are not large — the majority of infected animals survive — and that with 30 animals in each group the differences amount to six animals at most between the groups. This makes it difficult to know realistically what to make of the results. To address this problem, the authors would need to increase the power of their study by using larger sample sizes, which would show whether there is a significant increase in survival with drug treatment or not.

Indeed, UK funding agencies recently changed their animal experimental guidelines to reflect growing concerns that sample size is commonly too small in studies like this, which therefore may not have sufficient statistical power to detect real differences [1]. Appropriate sample sizes can be calculated based on the study design, and new tools are being developed to help researchers with this: one example is the Experimental Design Assistant, from the National Centre for the Replacement, Refinement & Reduction of Animals in Research [2], expected to launch later in 2015.
